# *Pistacia* Root and Leaf Extracts as Potential Bioherbicides

**DOI:** 10.3390/plants11070916

**Published:** 2022-03-29

**Authors:** Marta I. Saludes-Zanfaño, Ana M. Vivar-Quintana, María Remedios Morales-Corts

**Affiliations:** 1 Plant Production Group, Faculty of Environmental and Agricultural Sciences, Universidad de Salamanca, 37007 Salamanca, Spain; msz3223@usal.es; 2 Food Technology Group, Superior Polytechnic School of Zamora, Universidad de Salamanca, 49022 Zamora, Spain; avivar@usal.es

**Keywords:** allelopathy, weed density, root extract, leaf extract, germination rate, phenolic compounds

## Abstract

The allelopathic effect of pistachios was analyzed by field and laboratory tests. The parameters analyzed in the field trials were the biomass, weed density, weed diversity, and specific richness of the weed community. The studies were carried out in the area under the canopy and in the area beyond the influence of the pistachio tree, and the results obtained were compared. In the laboratory, germination bioassays were carried out on seeds of 11 weed species in root water extract, rhizosphere soil, and leaf water extract. The germination percentage, radicle elongation, epicotyl elongation, and germination index were determined. The results obtained show that significantly less biomass was present in the area under the influence of the trees, and fewer different weed species were detected in that area. In addition, germination bioassays showed that the aqueous leaf extract was a potent inhibitor of germination. The total content of flavonoids and phenols according to the organs (roots or leaves) was also studied. Extracts obtained from leaves showed higher concentrations of total phenols and also of flavones and flavanols than roots. Gallic acid, catechin, myricetin, and quercetin were identified in extracts obtained from both leaves and roots, while naringenin and rutin were identified only in the leaf extract. The presence of phenolic compounds in which allelopathic activity has been previously described and the results obtained in the trials seem to indicate that there is an allelopathic effect of the leaf extract, which could be used for weed control, thus facilitating ecological and/or sustainable management.

## 1. Introduction

Allelopathy is an interference mechanism in relation to the effect exerted on a plant owing to the action of chemical compounds released by another plant. The effects may occur either directly or indirectly and can be detrimental or beneficial to the recipient plant. Many studies have explored the allelopathic potential of plant species by focusing on the search for chemical compounds with herbicidal activity and also the effects of crops on weeds, on other crops, and on themselves [1]. In many studies of agricultural interest, allelopathic effects caused by the release of inhibitory chemicals emitted by a plant have been verified after observing the suppression of germination and the inhibition of the growth of other species. The ability of plants to suppress weeds is thus determined by crop allelopathy and competitiveness. Plant-to-plant interaction is a complex combination of competition for resources such as water, nutrients, and light, together with allelopathic interaction through allelochemicals [2]. It is therefore essential to eliminate competitive effects from experimental systems to identify the existence of allelopathy [3] under both field and laboratory conditions in order to demonstrate whether this factor can be used to control weeds in a crop system.

The usual effective methods of weed control in agriculture production (herbicide application and mechanical weeding) have many disadvantages, such as the evolution of herbicide resistance in weeds, the negative impacts of herbicides on the environment and human and animal health, the damage to the soil structure, and the enormous labor requirement. There is therefore a need to discover new herbicides since the number of herbicide-resistant weeds is increasing and conventional synthetic herbicides are becoming increasingly less effective against the resistant weed biotypes [4]. Weed population density and biomass production can be markedly reduced by using allelopathic plants [5]. In the southeastern region of Brazil, coffee fruit peel, which contains allelochemicals such as phenols, flavonoids, and caffeine, is often used as an organic amendment to agricultural practice in order to control weeds [6]. In this way, extracts of some allelopathic plants can be applied to manage some weeds by inhibiting germination and seedling growth [7], and there are some organic herbicides or plant growth inhibitors which have been manufactured from allelopathic plant materials [8,9].

The pistachio (*Pistacia vera*) is an important crop native to Central Asia grown for its edible nuts, mainly in Iran, the US, Turkey, and other warm temperate countries such as Spain. The *Pistacia* genus (Anacardiaceae) includes more than 12 types of deciduous trees, but among them, *P. vera* is the only commercially significant species producing large edible nuts, while *P. integerrima*, *P. atlantica*, *P. terebinthus*, etc., have economic application as rootstocks for the cultivars of *P. vera* [10]. According to the Agriculture Statistics drawn up by the Food and Agriculture Organization of the United Nations (FAO), 830,826 hectares of pistachios was cultivated in the world in 2020, with Iran’s share being 53% of the world’s planted area but the US accounting for more than 60% of the world production.

Alyousef and Ibrahim [11] verified the inhibitory effect of pistachio fruit skins and leaves on the growth of *Diplotaxis erucoides*, *Sonchus arvensis*, and *Papaver hibridum* in pot experiments. Taghvaeefard and Sadeghi [12] also found that leaf extracts of *Pistacia khinjuk* had an allelopathic effect on the germination of *Amaranthus retroflexus*. Recently, Tahir et al. [13] determined the phenolic composition of root and fruit extracts of *Pistacia atlantica* spp. *kurdica* by analyzing its inhibitory effect on the germination of certain weeds. Moreover, appreciations made by pistachio growers coincide in affirming that near the adult trees, the number of weeds developing is quite low. As Couceiro [14] reported, weed control tasks are considerably reduced after the trees are five years old. This could be the result not only of plant competition but also of the allelopathic effect caused by *P. vera* and its rootstocks. As a result, the study of this possible allelopathic effect is of great interest as a sustainable weed management method for this crop, since, according to Westwood et al. [15], research is needed on the crop–weed interaction from an allelopathic perspective for the new strategies of its control.

Phenolic compounds are among the most important and commonest plant allelochemicals in the ecosystem [16]. These compounds are secondary metabolites which plants synthesize naturally in order to develop, and their production is increased in stressful situations [17]. All these compounds are involved in different functions such as plant structure, pigmentation, pollination, resistance to pathogens and herbivores, and growth and development and are therefore essential for plant physiology. Insoluble phenolic compounds, bound to cellular components, contribute to the mechanical strength of cell walls and play a regulatory role in plant growth and morphogenesis, while compounds inside the cell are involved in the response to stress and pathogens [18]. Flavonoids are the most abundant phenolic compounds in plants; this group of compounds includes flavones, flavonols, flavonones, and dihydroflavonols. Among the non-flavonoids, phenolic acids are the main compounds in plants and present two distinctive carbon constitutive structures: the hydroxycinnamic and the hydroxybenzoic structure. There is a wide range of derivatives of these compounds as most are linked via ester, ether, or acetal bonds to plant structural components, larger polyphenols, or smaller organic molecules [19]. Within these compounds, hydroxybenzoic, vanillic, p-coumaric, and ferulic acids have been reported as common allelopathic compounds in rice [20]. Several phenolic acids (e.g., p-hydroxybenzoic, trans-p-coumaric, cis-p-coumaric, syringic, vanillic, and trans- and cis-ferulic acids) can be identified in allelopathic wheat cultivars [21]. Vanillic acid and p-coumaric acid have been found in a variety of plants and have demonstrated a potent allelopathic inhibition of other plants’ growth [22]. Different physiological mechanisms of action have been described for these compounds. Vanillic, ferulic, and p-coumaric acids have been shown to inhibit chlorophyll accumulation [23]. Syringic, caffeic, and p-protocatechuic acids have been found to be involved in the uptake of N, K, P, Fe, and Mo [24]. Benzoic, vanillic, cinnamic, and ferulic acids act on DNA and RNA synthesis [25]. According to Inderjit’s research on root exudates [26], primary and secondary compounds such as phenolic acids and their allelopathic effects have an impact on plant establishment. This experiment therefore aims to prove their involvement under natural conditions, which requires studies designed to be ecologically relevant.

To assess the allelopathic effects of pistachio root and leaf extracts and to investigate their use as potential bioherbicides, the objectives of the present study were: (i) to evaluate the effects of pistachio trees on the surrounding population of weeds in their environment; (ii) to test the phytotoxicity effects of root and leaf extracts on representative weeds; and (iii) to analyze the phenolic composition of aqueous and methanolic pistachio root and leaf extracts as the possible causes of the allelopathy.

## 2. Results

### 2.1. Field Study

During the 2020 and 2021 seasons, the weed population was evaluated in three different places under the canopies of trees over and under four years of age and also beyond the effect of pistachio trees.

Table 1 shows the results for weed presence and diversity indices under and beyond the influence of pistachio trees. It can be observed that beyond the area of influence of the trees, a higher biomass was generated (63.7 g m^−2^) than under the canopy, this value being significantly lower for adult trees (more than 4 years old). Plant density was likewise lower near the trees than beyond them. These facts may be due both to the direct competition of the trees with the adventitia vegetation and to allelopathic phenomena. The number of different species outside the area of influence (43.15) was significantly higher than that under the canopy (25.3 and 23.75 species under young and old trees, respectively). The reduction in the number of species could indicate an allelopathic effect which affects some species more than others. Fewer species were found under the canopy, especially in the case of >4-year-old trees, than beyond the influence of the trees. The pistachio trees therefore caused a reduction in the diversity of species, and for some of them, their presence was lower than outside their surrounding area. These results could support the production of allelopathic compounds by trees which selectively affect some adventitia.

In order to analyze the selective effect of pistachio trees on the weed population, the number of individuals for 15 representative weeds was counted. The species most frequently found in the orchard were *Bromus diandrus*, *Lolium rigidum*, *Epilobium brachycarpum*, *Conyza canadensis*, and *Datura stramonium*. The results are shown in Table 2. It is important to note that in 10 of the 15 species sampled (*Bromus diandrus*, *Conyza canadensis*, *Datura stramonium*, *Echium vulgare*, *Epilobium brachicarpum*, *Erigeron bonariensis*, *Lactuca sativa*, *Lolium rigidum*, *Scabiosa triandra*, and *Sinapis arvensis*), there was a significant reduction in the number of individuals per m^2^ under the canopy of adult trees in relation to the numbers found beyond the influence of the pistachio trees. The presence of young trees only led to a significant decrease in six species (*Bromus diandrus*, *Datura stramonium*, *Echium vulgare*, *Epilobium brachicarpum*, *Scabiosa triandra*, and *Sinapis arvensis*). The weed population of these six species was significantly reduced by the pistachio influence regardless of age. The greatest reduction in the number of plants found below adult trees compared with beyond their influence was obtained for *Datura stramonium*, *Sinapis arvensis*, *Bromus diandrus*, and *Echium vulgare*. Despite the lack of significant differences between the presence of the weeds studied near the influence of young trees and those more than four years old, there was a tendency for the number of plants of these species to decrease with the age of the pistachio trees, with the exception of *Bromus diandrus* and *Rumex acetosa*.

### 2.2. Germination Bioassays

With the aim of analyzing the effect of root and leaf water extracts in addition to rhizosphere soil on the germination and initial growth of 11 weeds, bioassays were carried out in Petri dishes (germination was not achieved for 4 weed species). This type of experiment investigates the allelopathic effect without taking into account the competitive effect which occurs in the field. The results are shown in Table 3.

The germination percentage (G%) of plants was negatively affected by the rhizosphere soil, especially in the case of *Solanum nigrum*, *Scabiosa triandra*, and *Rumex acetosa*, the figure for which was much lower than that of the control. Moreover, radicle length was lower than that of the control for *Sonchus asper*, *Sinapis arvensis*, *Solanum nigrum*, and *Scabiosa triandra* (*p* < 0.05). On the other hand, the rhizosphere soil improved the GI parameter (germination index with regard to the water control) in the case of *Bromus diandrus*, *Conyza canadensis*, *Lactuca serriola*, and especially *Centaurea melitensis*. The rhizosphere soil had a particularly negative effect on *Solanum nigrum*, lowering germination from 93.3% to 20%.

In the case of the root extract, no significant differences were found for the G% with regard to the control except for *Scabiosa triandra*. In this species, the germination was significantly lower than for the control. When germination was achieved, root extracts caused a significant reduction in the epicotyl length for *Lactuca serriola*. Moreover, the epicotyl length became significantly greater than that of the control for *Centaurea melitensis*, *Rumex acetosa*, and *Sinapis arvensis*. This effect also occurred in the radicle length of *Rumex acetosa*. *Scabiosa triandra*, *Sinapis arvensis*, and *Sonchus asper* all had shorter radicle lengths. Neither the root extract nor the rhizosphere soil influenced the germination or growth of the radicles or epicotyls in *Taraxacum officinale.*

The germination of the test plant species was severely inhibited by leaf extracts (Table 3). Germination was entirely prevented in *Echium vulgare*, *Rumex acetosa*, *Scabiosa triandra*, *Sinapis arvensis*, and *Taraxacum officinale* and caused a significant reduction in %G in the remainder of the species, with the exception of *Bromus diandrus*, in which the G% was similar to the control. Nevertheless, for *Centaurea melitensis*, *Conyza canadensis*, *Lactuca serriola*, *Solanum nigrum*, and *Sonchus asper*, when germination was achieved, the radicle length was significantly reduced by leaf extracts. In the same way, when germination was obtained by this medium, the epicotyl length was significantly reduced in the case of *Centaurea melitensis* and *Lactuca serriola. Scabiosa triandra* was found to be the most sensitive species by reducing the GI relating to the control in the three different bioassays. Although we did not find a statistically significant effect of the pistachio tree influence on the number of plants in the field for *Centaurea melitensis* and *Solanum nigrum*, the germination bioassays revealed a clear allelopathic effect as a result of not only the leaf extract but also the root extract. In general, the species most sensitive to the pistachio effect were *Lactuca serriola* and *Scabiosa triandra* both in the field and in vitro bioassays.

### 2.3. Determination of Total Phenolic Compounds and Flavonoids

Different photometric methods were used for the quantification of the three main groups of phenolic compounds (flavones and flavonols; flavanones and dihydroflavonols; and total phenols). The results (Table 4) show that all the extracts revealed the presence of compounds for all the phenolic groups analyzed. The methanol–water extracts showed higher concentrations of phenolic compounds than the water extracts. Total phenols, flavones, and flavonols were found in higher amounts in leaf extracts than in root extracts. In methanolic extracts, however, there were no significant differences between flavanones and dihydroflavonols, although there were considerable differences in the aqueous extract.

A total of six phenolic compounds were identified in the samples analyzed (Table 5). These compounds were identified by comparing their retention times and UV–Vis spectrum properties with those of reference standards. The identification was confirmed with the MS spectra. Among the phenolic acids, gallic acid was identified in leaves and roots regardless of the solvent used in the extraction. The highest concentrations of this compound were found in the aqueous extracts of leaves. Among the flavonoids, rutin, catechin, myricetin, quercetin, and naringenin were identified. Quercetin is the most abundant flavonoid in *Pistacia vera* leaves, with the highest amounts found in methanol–water extracts. Rutin and naringenin showed similar amounts regardless of the solvent used in the extraction. Myricetin and catechin showed a significantly higher concentration in the aqueous extracts, with the concentrations found being lower in the root extracts than in the leaf extracts. It was not possible to detect the presence of gallic acid and catechin in the aqueous root extracts.

## 3. Discussion

In this study, we obtained field results for weed presence and diversity indices under and beyond the influence of pistachio trees. Almost all the species found in the field have been included by Cirujeda et al. [27] in their list of weed species found in Spanish cereal fields, and our figures for richness from the area free of the effect of the trees are similar to those stated by these authors.

The pattern of the species richness of weed communities on European arable land is complex and is affected by several mutually correlated and often interacting factors. Differences in weed floras are largely attributable to management, which is partly related to crop-specific agricultural practices [28] and partly to broad-scale variation in environmental factors and general changes in the management of arable fields over the last five decades [29].

Because of the reduction in both the number of plants and their diversity, in addition to the changeover and the richness of species in our area of study with less richness under adult trees than beyond them, we can think of pistachio trees as producing allelopathic compounds which selectively affect certain adventitia rather than competition. In this sense, some authors [30] argue that the spatial distribution of crops and the distance between rows are of great importance in the regulation of weeds, not only owing to a competitive effect but also to the allelopathic effect which crop residues and soil exudates may cause. The results obtained in this study thus show a reduction in species richness, the number of species, density, and biomass owing to the age of the trees; the highest values for all these parameters correspond to the locations in the orchard which are beyond the influence of the trees. The selection of weed species found under adult pistachio trees suggests that the latter have an allelopathic effect. This fact was highlighted by Zamorano [31], who argued that competitiveness could be related to plant selection by allelopathy, which affects some species more than others. Furthermore, Pyšek et al. [29] pointed out that there is less competition between weeds and crops in nutrient-rich soils and irrigation systems, as is the case with the orchard studied. In this research, 10 of the 15 weed species considered (*Bromus diandrus*, *Conyza canadensis*, *Datura stramonium*, *Echium vulgare*, *Epilobium brachicarpum*, *Erigeron bonariensis*, *Lactuca sativa*, *Lolium rigidum*, *Scabiosa triandra*, and *Sinapis arvensis*) were found to be present with fewer individuals per m^2^ under the canopy of adult trees than beyond the influence of the pistachio trees. The greatest reduction was obtained for *Datura stramonium*, *Sinapis arvensis*, *Bromus diandrus*, and *Echium vulgare*. We also found that there was a tendency towards a reduction in the number of plants of the 15 weed species studied with the age of the pistachio tree, except for *Bromus diandrus* and *Rumex acetosa*. In this sense, Pardo-Muras et al. [32] indicated that gorse and scotch broom foliage added to the soil considerably reduced the dicotyledon biomass, except for *Solanum nigrum*. In accordance with these results, our study showed an important decrease in the number of individuals per m^2^ of the dicotyledon species and no reduction for *S. nigrum*.

Through germination bioassays, we determined that the leaf extracts showed a very clear decrease in the relative germination index. The leaf extract suppressed the seed germination of *Echium vulgare*, *Rumex acetosa*, *Scabiosa triandra*, *Sinapis arvensis*, and *Taraxacum officinale* and caused a very high reduction in the germination percentage of the remainder of the species, with the exception of *Bromus diandrus*, for which the germination percentage was similar to that of the control. Nevertheless, for *Centaurea melitensis*, *Conyza canadensis*, *Lactuca serriola*, *Solanum nigrum*, and *Sonchus asper*, when germination was achieved, the radicle length was significantly reduced by the leaf extracts. Similarly, in the case of germination in an aqueous leaf extract medium, the epicotyl length was significantly reduced in the case of *Centaurea melitensis* and *Lactuca serriola.* Other authors have pointed out that radicle elongation was greater than epicotyl elongation [33,34].

While the germination bioassay results from the leaf extracts show a very clear decrease in the relative germination index, root extracts gave different responses. Only in the case of *Lactuca serriola* did root extracts cause a significant reduction in the epicotyl length, and for *Scabiosa triandra*, *Sinapis arvenis*, and *Sonchus asper*, the radicle length was also reduced. In contrast, when germination was achieved for *Centaurea melitensis*, *Rumex acetosa*, and *Sinapis arvensis*, the length of the epicotyls increased, as did the length of the radicles for *Rumex acetosa*. Similar results were obtained for the shoot length of wild asteraceae using a low aqueous extract concentration of *Parthenium hysterophorus* leaves [33]. The sensitivity to allelochemicals and the extent of inhibition differ among species and organs of the test species according to numerous authors [33,35]. It is therefore probable that low concentrations of allelopathic chemicals in root extracts encourage the favorable growth of certain organs of several species considered in this research. Furthermore, different evolutionary histories of invasive species are generally thought to have conferred diverse mechanisms of resistance on allelopathic chemicals [36,37].

Bioassay results may indicate a particular effect on asteraceae weeds, which should be studied in future research. Leaf extracts may have a clear effect on dicotyledon weed species, possibly causing the same effects found by Pardo-Muras et al. [32] in *Ulex europaeus*. These authors found that water-soluble compounds from foliage such as terpenoids, phenolic acids, and flavonoids caused the most relevant bioherbicidal effects.

Thair et al. [13] tested the allelopathic effect of fruit and root extracts of *Pistachia atlantica* on two weed species (Narbon vetch and wild mustard) by bioassay germination; it was found that these extracts significantly inhibited the germination and growth of both species. Our results coincide with theirs, as in *Sinapis arvensis* (wild mustard), the effect of the leaf extract was the same as that obtained with the extract of fruit skins. In general, our results are also consistent with those achieved with extracts of *Pistacia khinjuk* leaves [12]. For *Lactuca serriola*, we found a control germination ratio of 60%, which fell to 3.33% for leaf extracts in which the catechin content was very high.

In accordance with our results, Maharjan et al. [33] also pointed out that the highest germination inhibition occurs in aqueous leaf extracts compared to the roots. In their study, these authors showed that a concentrated aqueous extract of leaves of *Parthemium histerophorus* inhibited the seed germination and seedling growth of invasive asteraceae weeds such as *Ageratina adenophora* and *Artemisia dubia* and suggested that this plant could be exploited as a natural herbicide source owing to its water-soluble allelochemicals such as phenolic acid. They indicated strong inhibition in the seed germination of crucifers and asteraceae. Rajendiran [38] detected important problems in mitosis owing to the effect of the aqueous extract of *P. histerophorus*. This could only occur when certain allelochemicals in the leaf extract prevented the embryo from growing or caused its death.

In relation to our study of the phenolic compounds present in roots and leaves, it was observed that the concentration in water extracts was lower than in methanol–water extracts, depending on the solvent used for extraction. The relative proportions of each group of compounds were, however, the same regardless of the solvent used. This is due to the strong influence of the polarity of the solvent used in the extraction process on the ability to extract such compounds [39]. Phenolic compounds are more soluble in ethanol–water mixtures than in water alone [40].

The results we obtained from the leaves are in keeping with those of previous studies by Yemmen et al. [41] and Garofulić et al. [42] on the total phenolic content of leaves of *Pistacia lentiscus*. All the results obtained in relation to the total phenolic content are within the range described by other authors, with concentrations of 68.23 mg GAE/g in *Pistacia atlantica* [43] and 428.10 mg GAE/g in *Pistacia vera* [39], showing higher values than those described for flavonoids of between 19.162 mg CE/g [44] and 64.38 mg CE/g [39]. In the case of roots, these results agree with those found in methanolic extracts in the work carried out by Amel et al. [45], in which the roots of *Pistacia lenticus* showed lower concentrations of both phenolic compounds and flavonoids than the leaves. These differences can be explained by the non-uniform distribution of these types of compounds at the cellular and subcellular plant tissue levels [18]. In the case of roots, the values in this study are higher than those described by other authors for both water and methanol–water extracts. Differences between other pistachio parts analyzed have also been described for both the quantitative content and the qualitative chemical profile of phenolic compounds [42,46]. Differences have even been described between male and female leaves [39]. The presence of phenolic compounds in root exudates has been extensively described, with phenolic acids being the most abundant compounds [47]. The substances extracted by water closely coincide with the amounts of compounds present in the aqueous extracts of our tests. Aqueous extracts obtained from leaves showed higher concentrations of total phenols as well as flavones, flavanols, flavanones, and dihydroflavanols, than aqueous extracts from roots. These results could explain that the aqueous leaf extract suppressed the germination of five weed species and caused a very high reduction in the germination percentage in the remainder except for the monocotyledonous species. The different response in the bioassays of leaf extracts in relation to root extracts could be related to the amount of phenolic compounds.

The individual compounds identified in this study are consistent with those described in papers previously published. In *Pistacia atlantica*, several authors have thus reported the presence of gallic acid and rutin in leaves [48,49]. Yousfi et al. [50] identified gallic acid and naringenin in methanolic extracts of leaves, while Toul et al. [44] identified gallic acid, rutin, catechin, quercetin, and naringenin in leaves. In *Pistacia vera*, Boumaiza et al. [39] reported the presence of gallic acid, rutin, catechin, myricetin, quercetin, and naringenin in leaves. In *Pistacia lenticus*, Mehenni et al. [51] identified the presence of gallic acid, while in studies carried out on leaves, Elez Garofulić et al. [42] found gallic acid to be the most abundant phenolic acid, with catechin standing out as the most important flavonol among the flavonoids.

In the case of roots, fewer studies have analyzed the individual phenolic composition. Toul et al. [44] reported that fewer phenolic compounds were detected in the roots than in the leaves of *Pistacia atlantica* and, as in our study, did not detect rutin in the roots.

As for the concentrations at which these compounds are present, the literature available shows a wide range. Those of gallic acid described for leaves range from 8.51 µg/g dw [48] to 700 µg/g dw [44]. The concentrations found in this study are within the figures mentioned by other authors. In addition, the different solvents used in the extraction of the individual compounds have also been shown to have a great influence on the concentrations of the compounds found. The methanol–water extracts showed a higher concentration of quercetin, while the water extracts showed higher concentrations of gallic acid and catechin. These results are in agreement with the solubility of these compounds, as gallic acid and catechin are water-soluble compounds, while quercetin has low solubility in water. The type of extraction and the part of the plant used for the study have a great influence on the compounds identified and their concentration in studies carried out on different subspecies of *Pistacia atlantica* [52]. The type of extraction used seems to have more influence on the compounds extracted from the roots [44]. This is in keeping with the results of this study.

The allelopathic activity of the compounds identified in this study has already been reported. Fernández-Aparicio et al. [53] thus described the reduction in root growth and haustorium induction as allelopathic effects caused by quercetin. These authors associated the inhibition of root growth with the cessation of root elongation but did not observe browning or any other visible signs of toxicity in root tissue. Rudrappa et al. [54] identified gallic acid as the compound responsible for the root phytotoxicity of *P. australis*. Gallic acid causes the complete collapse of the root architecture owing to the high levels of reactive oxygen species (ROS) which it generates in the roots of treated plants. Catechin has been reported to show broad-spectrum allelochemical activity against several plant species [55]. This compound reacts with metals occurring naturally in the soil to form catechin–metal complexes; for this reason, the allelopathic effects of catechin are variable and difficult to determine [56]. Catechin was considered to act as an allelochemical on invasive plant species by Kato-Noguchi [3]. These data are in keeping with the affirmation of the inhibitory activity of catechin against the growth of *Lactuca sativa* reported by this author. Myricetin together with quercetin has shown inhibitory effects on lettuce radicle growth [57]. In addition, their ability to inhibit the germination and root growth of *Glycine max*, *Lepidium sativum*, and *Raphanus sativus* has been described [58]. Regarding naringenin, Deng et al. [59] showed that it inhibited the seedling growth of *Oryza sativa*, *Zea mays*, and *Echinochloa oryzicola*. In addition, phenolic acids can increase the activity of phenylalanine ammonialyase (PAL) and B-glucosidase while reducing the activity of phenol-B-glucose transferase, thus inhibiting root growth [5]. Some authors [60,61] have demonstrated that most phenolic allelochemicals can influence endogenous hormone levels, mainly by stimulating IAA oxidase activity and thus reducing IAA levels, which produces and inhibits seedling growth. Moreover, Lin et al. [62] argued that caffeic acid, gallic acid, and phenols regulate phenylalanine metabolism by suppressing the activities of PAL and cinnamic acid-4-hydroxylase. The allelopathic effects found in this study were probably caused by the high concentration of gallic acid, catechin, myricetin, and quercetin found in the aqueous leaf extract.

Adding pistachio leaves to the soil of orchards could therefore be an interesting method of integrated weed management in order to reduce weed populations in orchards. This type of management has been indicated for other species such as coffee; in coffee plantations, not only leaves but also fruit peel is often used to control weeds [5]. Kerman leaves could thus be used as a bioherbicide since, according to Pardo-Muras et al. [32], allelopathic biomass could represent a real alternative which is more environmentally friendly and safe than the application of synthetic herbicides. According to Puig et al. [1], the release of volatile and water-soluble allelochemicals from *Eucalyptus globulus* leaves was sustained after adding foliage for more than one month. As a follow-up to our results, it may be possible to manufacture an organic herbicide from pistachio leaf water extracts, but further studies are needed to analyze the correct concentration and the effect on important referent crops. As also pointed out by some authors [1,32], organic herbicides should be used as pre-emergence weed control. It therefore follows that practical field studies are also necessary to support the use of *Pistacia vera* leaf extracts as a bioherbicide.

## 4. Materials and Methods

### 4.1. Field Study

#### 4.1.1. Location and Orchard Characteristics

This study was carried out in a five-year-old pistachio orchard (with coexisting trees of different ages) with drip irrigation located in the village of Parada de Rubiales (Salamanca-Castilla y León-Spain) (41°9′18.02″ N, 5°26′50.24″ W, 844 m.a.s.l.). The density was 400 trees per hectare. The soil was determined as a calcium Cambisol, with a loamy-clay-sandy texture and a pH of 8.1; the annual rainfall is 380 mm; and the average annual temperature is 11.7 °C. The female trees correspond to the most important cultivated variety in the world, i.e., ‘Kerman’ [63], with ‘UCB1’ as a rootstock (*Pistacia integerrima* × *Pistacia atlantica*), which is considered the best commercial rootstock for irrigation conditions [64]. No herbicides were used in the orchard, with the weed management being exclusively tillage.

#### 4.1.2. Parameters Analyzed

With the aim of analyzing the effect of the roots and leaves on weed presence and growth, the weed population in three different locations of the orchard was studied during the 2020 and 2021 seasons:

Location one: under the canopy of two- and three-year-old trees;

Location two: under the canopy of trees more than four years old;

Location three: beyond the influence of the trees (inter-row areas and boundaries).

The orchard was divided into 6 plots of 1500 m^2^. Four trees of each different age were randomly selected per plot. The plant biomass was cut with scissors under the canopies and beyond the influence of the trees into 0.5 × 0.5 m^2^ quadrates. A total of 24 samples per location and season were taken.

The weeds collected were identified based on genus and species, and the number of individuals of each weed species was counted. To evaluate weed presence and biodiversity, some indices were determined including the total biomass (oven drying at 65 °C for 48 h), the number of different species, the plant density, and the species richness (Margalef index). The Margalef index (d) is calculated as follows: d = (S−1)/lnN, in which S is the number of species, and lnN is the natural logarithm of the total number of individuals in the sample.

Moreover, with the objective of evaluating the influence of the pistachio trees on 15 frequent weed species (*Bromus diandrus, Centaurea melitensis, Conyza canadensis, Datura stramonium, Echium vulgare, Epilobium brachicarpum, Erigeron bonariensis, Lactuca serriola, Lolium rigidum, Rumex acetosa, Scabiosa triandra, Sinapis arversis, Solanum nigrum, Sonchus asper,* and *Taraxacum officinale*), their presence under and beyond the influence of the trees was determined using samples of 0.5 × 0.5 m^2^ quadrates (the total plant numbers of each species). The weed species were selected as common and invasive in the irrigation crop system.

### 4.2. Germination Bioassays

#### 4.2.1. Preparation of Root and Leaf Pistachio Aqueous Extracts

Fresh roots and mature leaves were finely minced soon after collection (Molineux mincer AD560120). In all cases, 250 g of plant material was immersed in 1000 mL distilled water in a 2 L Erlenmeyer flask, which was equivalent to the percentage suggested by Taghvaeefard and Sadeghi [12]. Flasks were left in the dark for 24 h at room temperature and gently soaked every 6 h. A cellulose membrane (0.45 m pore size) was used to vacuum filter the aqueous extracts. The extracts were then divided into two groups: those which were used immediately for bioassays, and those which were frozen at −18 °C until needed.

#### 4.2.2. Bioassay Procedure

During the year 2020, seeds from 15 different weed species were collected in the orchard investigated. Different circumstances were attempted in order to accomplish germination control, and eventually a minor mechanical scarification was required; however, the species *Datura stramonium*, *Erigeron bonariensis*, *Epilobium brachicarpum*, and *Lolium rigidum* did not germinate. The bioassays were then carried out in 9 cm-diameter Petri dishes with Whatman 3 paper inside, with 10 seeds of each species per dish. The following treatments were used: aqueous extract of roots, rhizosphere soil, and aqueous extract of mature leaves, with distilled water as a control. Each Petri dish held 10 mL of the relevant extract and 30 mL of rhizosphere soil. The plates were maintained at 20 °C in the dark for 10 days, as described by Tahir et al. [13] with minor modifications (except for *Rumex acetosa*, which was kept for 30 days adding twice the corresponding extract). Four replications per treatment were carried out, and the test was repeated twice. The parameters determined were: the germination percentage (G%), radicle length, epicotyl length, and germination index with respect to the control (GI), following the Zucconi methodology [65].

IG = (PGR × ERR × 100)

with PGR being (%G _extract_/%G _control_) and ERR being (radicle length _extract_/radicle length _control_).

### 4.3. Extraction and Determination of Phenolic Compounds

#### 4.3.1. Extract Preparation

Two samples (roots and leaves) were extracted following the methodology described by Betances-Salcedo et al. [66] with some modifications. An amount of 1 g of each sample (root or leaf) was macerated with 20 mL of water for 8 min in an XUBA1 ultrasonic bath (Grant). The liquid was then centrifuged and collected in the freezer (−18 °C) for further analysis. To obtain the methanolic extract, we proceeded in the same way but used a methanol–water mixture (85:15) for the extraction.

#### 4.3.2. Determination of Total Phenols

The method described by Boumaiza et al. [39] was used with minor modifications. In a 25 mL volumetric flask, 0.5 mL of phenolic extract was placed together with 0.5 mL of Folin–Ciocalteu reagent and 10 mL of 7.5% Na_2_CO_3_ and completed with distilled water. The absorbance was measured at 750 nm using a Shimadzu spectrophotometer after 1 h of rest at room temperature in the dark (Columbia, MD, USA). The quantity was calculated using a gallic acid standard curve and is given in milligrams of gallic acid per gram of dry weight (dw).

#### 4.3.3. Determination of Flavone and Flavonol Content

The total content of flavones and flavonols was estimated by the colorimetric method based on the formation of the aluminum chloride complex described by Valencia et al. [67]. In a 25 mL volumetric flask, 2 mL of phenolic extract was placed and 500 µL of 5% AlCl was added and made up to the mark with 96% ethanol. The mixture was kept for 30 min (at room temperature) in the dark. The absorbance was measured at 425 nm in a spectrophotometer. Quercetin was used to construct the calibration curve. The results are expressed as milligrams of quercetin per gram of dried weight.

#### 4.3.4. Determination of Flavanones and Dihydroflavonols

The content of flavanones and dihydroflavonols was obtained spectrophotometrically according to the method described by Popova et al. [68] with slight modifications. An aliquot (1 mL) of phenolic extract was heated at 50 °C for 50 min with 2 mL of DNP (2,4-dinitrophenylhydrazine) solution. After allowing 10 mL to cool to ambient temperature, 10% potassium hydroxide in methanol (*w*/*v*) was added. At 486 nm, the absorption was measured. The results are given in milligrams of pinocembrin per gram of dry weight.

#### 4.3.5. Identification and Quantification of Individual Phenolic Compounds

The phenolic composition of the samples (roots and leaves) was analyzed using the method proposed by Vivar-Quintana et al. [69], with some modifications. HPLC analyses were carried out on a 1100 modular system Agilent Technologies model (Agilent Technologies, Palo Alto, CA, USA) consisting of a vacuum degasser, a quaternary pump, an autosampler, a thermostated column, and a diode array detector (DAD). A Hypersil ODS C18 column (250 mm × 4.6 mm) was used for HPLC analysis. The mobile phase consisted of 0.1% formic acid in H_2_O (A) and acetonitrile (B). Diode array acquisition was performed in the range of 220–550 nm, and chromatograms were integrated at 280 and 330 nm depending on the compounds analyzed. Two injections were performed for each sample. The gradient elution was modified as follows: 0–14 min 15% B, 14–38 min from 15% to 25% B, 38–45 min from 25% to 30% B, 45–49 min from 30% to 40% B, and 49–54 min from 40% to 50% B. The flow rate was 1 mL/min. The column temperature was set at 30 °C. The single injection volume was 30 μL. Quantification was carried out using a calibration curve for each of the compounds identified: gallic acid, myricetin, rutin, catechin, and quercetin 3-glucoside. The concentration of naringenin was calculated using the quercetin 3-glucoside calibration curve.

### 4.4. Statistical Analysis

Statistical processing of the data was carried out using IBM-SPSS Statistics 26 software (IBM, Chicago, IL, USA). The differences between treatments were determined using ANOVA, and when significant differences were found, the Tukey range test (*p* < 0.05) was applied as a post hoc analysis. The germination parameter, which was assessed in percentages in this study, was previously arcsine transformed using Bartlett’s equation [70]:

$$\arcsin transf=2\times \left(\sin^{-1}\left(\sqrt{X\%}\right)\right)$$

The findings of this parameter are given as percentages to make them easier to understand, but the statistical differences were examined using the arcsine transformation values.

## 5. Conclusions

Pistachio trees have a reducing effect on biomass and species diversity in their area of influence. The aqueous extract of pistachio leaves was shown to inhibit the germination of almost all the weed species tested. The total phenol, flavone, and flavanol concentrations in leaf extracts were higher than those in root extracts. The high content of phenolic compounds in leaves, mainly gallic acid, catechin, myricetin, and quercetin, supports the allelopathic effects on the weed species analyzed. This allelopathic effect of the aqueous leaf extract could be used for weed control by facilitating ecological and/or sustainable management. Based on these findings, pistachio leaf extracts could be used to create an organic herbicide. Knowledge of allelopathic effects may be crucial in the face of biological weed control which allows the elimination of or reduction in the use of chemical pesticides.

## Figures and Tables

**Table 1:** Weed presence and diversity indices under and beyond the influence of pistachio trees. All values represent the mean and ± SD.

Place of Sampling	Biomass (g.m.s./m^2^)	Density (pl/m^2^)	N° Species/m^2^	Richness of Species (Margalef’s Index)
>4-year-old trees	32.6 ± 4.8 ^c^	100.1 ± 12.9 ^b^	23.75 ± 1.8 ^b^	0.71 ± 0.1 ^b^
2–3-year-old trees	57.5 ± 6.2 ^b,c^	120.9 ± 20.7.8 ^b^	25.3 ± 2.4 ^b^	0.77 ± 0.1 ^b^
Beyond influence	63.7 ± 4.6 ^a^	241.14 ± 25.8 ^a^	43.15 ± 3.2 ^a^	1.45 ± 0.2 ^a^

ANOVA, analysis of variance; a–c post hoc: Tukey HSD test. Different letters in the same column indicate statistically significant differences; *p* < 0.05; *n* = 24.

**Table 2:** Presence of different weed species under pistachio trees and beyond their influence. All values represent the mean and ± SD.

Weed Species	Under Canopy Young Trees (Individuals/m^2^)	Under Canopy > 4-Year-Old Trees (Individuals/m^2^)	Beyond Influence of Pistachio Trees (Individuals/m^2^)
*Bromus diandrus*	10.00 ± 7.9 ^b^	22.14 ± 12.2 ^b^	122.78 ± 18.2 ^a^
*Centaurea melitensis*	1.67 ± 1.2 ^a^	1.00 ± 2.2 ^a^	5.00 ± 2.1 ^b^
*Conyza canadensis*	24.84 ± 4.2 ^a,b^	18.24 ± 6.2 ^b^	37.78 ± 3.2 ^a^
*Datura stramonium*	8.5 ± 4.5 ^b^	4.50 ± 2.25 ^b^	21.2 ± 6.25 ^a^
*Echium vulgare*	2.00 ± 1.8 ^b^	1.00 ± 2.3 ^b^	5.00 ± 1.1 ^a^
*Epilobium brachycarpum*	30.00 ± 6.2 ^b^	20.65 ± 5.2 ^b^	45.00 ± 3.2 ^a^
*Erigeron bonariensis*	7.78 ± 2.2 ^a,b^	6.11 ± 1.2 ^b^	12.50 ± 4.2 ^a^
*Lactuca serriola*	7.95 ± 2.95 ^a,b^	6.74 ± 2.15 ^b^	12.38 ± 2.31 ^a^
*Lolium rigidum*	62.14 ± 26.2 ^a,b^	26.05 ± 20.2 ^b^	75.00 ± 18.2 ^a^
*Rumex acetosa*	7.78 ± 3.2 ^a^	8.33 ± 2.2 ^a^	10.50 ± 4.2 ^a^
*Scabiosa triandra*	5.71 ± 1.2 ^b^	5.00 ± 1.6 ^b^	13.00 ± 3.3 ^a^
*Sinapis arvensis*	1.67 ± 2.4 ^b^	1.00 ± 1.2 ^b^	7.14 ± 2.2 ^a^
*Solanum nigrum*	2.50 ± 2.2 ^a^	1.25 ± 2.2 ^a^	5.00 ± 3.3 ^a^
*Sonchus asper*	7.00 ± 4.6 ^a^	6.67 ± 3.2 ^a^	10.63 ± 5.2 ^a^
*Taraxacum officinale*	3.00 ± 1.2 ^a^	1.00 ± 2.2 ^a^	2.50 ± 1.7 ^a^

ANOVA, analysis of variance; ^a,b^ post hoc: Tukey HSD test. Different letters in the same row indicate statistically significant differences; *p* < 0.05; *n* = 24.

**Table 3:** Effect of pistachio root and leaf extracts on germination and seedling parameters of selected weeds. All values represent the mean and ± SD.

Weeds	Parameters	Water Control	Root Extract	Rhizosphere Soil	Leaf Extract
*Bromus diandrus*	G (%)	33.33 ± 10.3 ^a^	20 ± 10.0 ^a^	36.67 ± 15.3 ^a^	33.33 ± 5.8 ^a^
	Radicle length (mm)	2.99 ± 1.0 ^a,b^	4.47 ± 1.3 ^a^	3.87 ± 3.8 ^a^	0.72 ± 0.4 ^b^
	Epicotyl length (mm)	0.69 ± 0.5 ^a^	1.15 ± 0.9 ^a^	1.02 ± 1.4 ^a^	0.64 ± 0.5 ^a^
	GI (%)		89.71	142.40	24.08
*Centaurea melitensis*	G (%)	61.67 ± 29.9 ^a^	20 ± 11.8 ^a,b^	66.67 ± 20.8 ^a^	16.67 ± 5.8 ^b^
	Radicle length (mm)	1.69 ± 0.7 ^b^	2.1 ± 0.9 ^a,b^	3.33 ± 1.3 ^a^	0.14 ± 0.1 ^c^
	Epicotyl length (mm)	1.18 ± 0.5 ^c^	2.23 ± 0.7 ^b^	3.24 ± 1.16 ^a^	0.12 ± 0.1 ^d^
	GI (%)		40.30	213.02	2.24
*Conyza canadensis*	G (%)	76.67 ± 8.2 ^a^	66.67 ± 15.3 ^a^	86.87 ± 5.8 ^a^	10 ± 0.8 ^b^
	Radicle length (mm)	0.35 ± 0.1 ^a^	0.32 ± 0.1 ^a^	0.38 ± 0.1 ^a^	0.1 ± 0.0 ^b^
	Epicotyl length (mm)	0.35 ± 0.2 ^ab^	0.57 ± 0.2 ^b^	0.85 ± 0.3 ^a^	0.13 ± 0.1 ^c^
	GI (%)		79.50	123.02	3.73
*Echium vulgare*	G (%)	23.33 ± 11.1 ^a^	6.67 ± 4.3 ^a^	6.67 ± 5.2 ^a^	
	Radicle length (mm)	1.52 ± 1.5 ^ab^	1.85 ± 1.8 ^a,b^	2.05 ± 1.1 ^a^	N.G.
	Epicotyl length (mm)	0.53 ± 0.5 ^b^	1.2 ± 1.2 ^a,b^	2.7 ± 0.5 ^a^	
	GI (%)		35,03	38,81	
*Lactuca serriola*	G (%)	60.00 ± 26.0 ^a^	13.33 ± 5.2 ^a,b^	63.33 ± 13.7 ^a^	3.33 ± 5.8 ^b^
	Radicle length (mm)	1.72 ± 1.3 ^a^	1.02 ± 1.0 ^a,b^	1.87 ± 0.6 ^a^	0.2 ± 0.0 ^b^
	Epicotyl length (mm)	1.11 ± 0.9 ^b^	0.52 ± 0.5 ^c^	2.78 ± 0.4 ^a^	0.4 ± 0.0 ^c^
	GI (%)		13.18	114.75	0.65
*Rumex acetosa*	G (%)	33.33 ± 23.1 ^a^	20.00 ± 10.0 ^a^		
	Radicle length (mm)	1.86 ± 2.1 ^b^	5.02 ± 0.9 ^a^	N.G.	N.G.
	Epicotyl length (mm)	1.13 ± 1.2 ^b^	3.17 ± 0.5 ^a^		
	GI (%)		161.95		
*Scabiosa triandra*	G (%)	56.67 ± 23.5 ^a^	20.00 ± 10.0 ^b^	16.67 ± 11.5 ^b^	
	Radicle length (mm)	3.18 ± 1.6 ^a^	1.57 ± 1.1 ^b^	0.98 ± 1.1 ^b^	N.G.
	Epicotyl length (mm)	1.24 ± 0.6 ^a^	0.58 ± 0.8 ^a,b^	1.4 ± 1.0 ^a^	
	GI (%)		17.42	9.07	
*Sinapis arvensis*	G (%)	41.67 ± 27.9 ^a^	46.67 ± 41.6 ^a^	26.67 ± 11.5 ^a^	
	Radicle length (mm)	1.60 ± 0.7 ^a^	1.27 ± 0.4 ^b^	0.95 ± 0.5 ^b^	N.G.
	Epicotyl length (mm)	1.96 ± 0.5 ^b^	2.52 ± 0.5 ^a^	1.91 ± 0.8 ^b^	
	GI (%)		88.90	38.00	
*Solanum nigrum*	G (%)	93.33 ± 10.1 ^a^	36.67 ± 42.2 ^a,b^	20 ± 15.5 ^b^	13.33 ± 11.5 ^b^
	Radicle length (mm)	1.43 ± 0.6 ^a^	1.05 ± 0.3 ^a,b^	0.55 ± 0.3 ^b^	0.45 ± 0.17 ^b^
	Epicotyl length (mm)	2.11 ± 2.1 ^a^	1.33 ± 0.5 ^a^	0.25 ± 0.2 ^a^	0.15 ± 0.13 ^a^
	GI (%)		28.85	8.24	4.49
*Sonchus asper*	G (%)	43.33 ± 26.0 ^a,b^	31.67 ± 18.3 ^b^	26.67 ± 10.3 ^b^	3.33 ± 5.8 ^c^
	Radicle length (mm)	1.93 ± 0.8 ^a^	1.07 ± 0.3 ^b^	0.95 ± 0.3 ^b^	0.90 ± 0.1 ^b^
	Epicotyl length (mm)	1.86 ± 1.0 ^b^	1.76 ± 0.9 ^b^	2.65 ± 0.7 ^a^	2.1 ± 0.1 ^b^
	GI (%)		40.52	30.30	3.58
*Taraxacum officinale*	G (%)	76.67 ± 25.8 ^a^	93.33 ± 8.2 ^a^	70 ± 35.2 ^a^	
	Radicle length (mm)	0.86 ± 0.5 ^a^	0.96 ± 0.5 ^a^	0.82 ± 0.5 ^a^	N.G.
	Epicotyl length (mm)	2.29 ± 1.3 ^a^	2.66 ± 0.9 ^a^	2.61 ± 1.3 ^a^	
	GI (%)		135.88	87.05	

ANOVA, analysis of variance; ^a–c^ post hoc: Tukey HSD test. Different letters in the same row indicate statistically significant differences; *p* < 0.05; N.G. = no germination.

**Table 4:** Total flavonoid and phenolic contents (mg/g dw) according to the organs (roots or leaves) and extraction methodology (methanol–water extract or water extract). All values represent the mean of duplicate determinations ± SD.

	Methanol–Water Extract		Water Extract	
	Leaf	Root	Leaf	Root
Total phenols ^1^	127.85 ± 30.81 ^a^	68.00 ± 8.72 ^a,b^	45.28 ± 17.44 ^b^	13.24 ± 0.02 ^b^
Flavones and flavonols ^2^	49.71 ± 1.89 ^a^	13.72 ± 9.68 ^b,c^	33.01 ± 0.47 ^a,b^	1.77 ± 0.79 ^c^
Flavanones and dihydroflavonols ^3^	27.44 ± 0.18 ^a^	25.01 ± 1.24 ^a^	26.49 ± 4.25 ^a^	10.79 ± 1.08 ^b^

ANOVA, analysis of variance; ^a–c^ post hoc: Tukey HSD test. Different letters in the same row indicate statistically significant differences; *p* < 0.05. ^1^ Expressed as gallic acid equivalent. ^2^ Expressed as rutin equivalent. ^3^ Expressed as pinocembrin equivalent.

**Table 5:** Concentration of phenolic compounds (expressed as µg/g dw) according to the organs (roots or leaves) and extraction methodology (methanol–water extract or water extract).

	Methanol–Water Extract		Water Extract	
	Leaf	Root	Leaf	Root
Gallic acid	63.03 ± 0.02 ^b^	30.27 ± 2.63 ^c^	205.55 ± 2.42 ^a^	60.61 ± 2.08 ^b^
Rutin	80.73 ± 0.26 ^a^	ND	85.37 ± 9.87 ^a^	ND
Catechin	91.77 ± 0.75 ^b^	17.02 ± 9.31 ^c^	113.95 ± 2.49 ^a^	27.27 ± 1.71 ^c^
Myricetin	83.23 ± 1.30 ^b^	1.75 ± 2.42 ^c^	140.17 ± 1.83 ^a^	ND
Quercetin	263.24 ± 3.57 ^a^	1.66 ± 2.32 ^c^	158.76 ± 1.74 ^b^	ND
Naringenin	1.23 ± 0.07 ^a^	ND	0.86 ± 0.74 ^a^	ND

ANOVA, analysis of variance; ^a–c^ post hoc: Tukey HSD test. Different letters in the same row indicate statistically significant differences; *p* < 0.05; ND: not detected.

## Data Availability

Not applicable.
